# A long-term siRNA strategy regulates fibronectin overexpression and improves vascular lesions in retinas of diabetic rats

**Published:** 2011-12-06

**Authors:** Sumon Roy, Sigrid Nasser, Melissa Yee, Dana T. Graves, Sayon Roy

**Affiliations:** 1 Department of Medicine and Ophthalmology, Boston University School of Medicine, Boston, MA; 2 Department of Periodontics, University of Pennsylvania School of Dental Medicine, Philadelphia, PA

## Abstract

**Purpose:**

A sustained gene modulatory strategy is necessary for regulating abnormal gene expression in diabetic retinopathy, a long-term complication. We investigated the efficacy of a small interference RNA (siRNA) strategy in mediating the long-term downregulatory effect of fibronectin (FN) overexpression in vivo.

**Methods:**

Streptozotocin-induced diabetic rats were intravitreally injected with 3 µM of FN-siRNA at six week intervals over a period of 4.5 months. Retinal FN protein expression, vascular basement membrane (BM) thickness, and retinal vascular cell loss were assessed by western blot, electron microscopy, and retinal trypsin digest, respectively.

**Results:**

Retinal FN expression and BM thickness were significantly increased in diabetic rat retinas compared to those in non-diabetic control rats (188±14.2% of control versus 100±7.4% of control, p<0.002; 72.5±5.0 nm versus 51.5±4.8 nm, p<0.001, respectively). FN-siRNA treatment reduced FN overexpression and BM thickening (145±19.9% of control and 56.4±2.8 nm, respectively) and significantly reduced the number of acellular capillaries (35%) and pericyte loss (55%) compared to those of untreated diabetic eyes.

**Conclusions:**

These findings suggest that BM thickening is an important target for preventing vascular cell loss in a diabetic retina, and that the siRNA approach could be useful for long-term gene modulation in diabetic retinopathy.

## Introduction

Retinal capillary basement membrane (BM) thickening is closely associated with the development of vascular lesions in diabetic retinopathy, a long-term complication of diabetes. The detrimental effects of BM thickening in promoting the development of vascular lesions in diabetic retinopathy are beginning to be established. Studies indicate that BM thickening primarily develops from a process involving hyperglycemia-induced excess synthesis of BM components, such as fibronectin (FN), collagen type IV, and laminin [1–4]. The abnormal thickening of BM in the retinal capillaries is a long-lasting lesion of diabetic retinopathy and appears to promote other characteristic lesions, including acellular capillaries and pericyte loss, vascular leakage, and a disruption of the overall vascular homeostasis associated with diabetic retinopathy [1,5,6]. An effective long-term strategy to block retinal capillary BM thickening could be useful as a therapeutic intervention for preventing the damaging lesions resulting from diabetic retinopathy.

The role of FN overexpression and BM thickening has recently been examined in the retinal and glomerular capillaries of diabetic rats using antisense oligonucleotides [7]. The study demonstrated that tight glycemic control downregulates FN overexpression and reduces vascular BM thickening in retinal and renal tissues [7]. Previously, we have shown that among various BM components, downregulation of FN expression alone significantly reduces BM thickening and inhibits the development of acellular capillaries and pericyte loss in the retinas of galactose-fed rats [6]. Furthermore, the reduction of FN overexpression using siRNA has indicated improved cell monolayer permeability (Toshi siRNA). Overall, these findings indicate the importance of FN in mediating the barrier characteristics of the BM structure, and suggest that a strategy for reducing FN overexpression could be beneficial for improving retinal vascular lesions.

We previously demonstrated that antisense oligonucleotides could reduce BM thickening by lowering the expression of BM components [6], and that downregulation of BM components in the retina have beneficial effects in inhibiting blood retinal barrier breakdown [1]. Our findings proved that a thickened retinal capillary BM could be targeted for therapeutic intervention and that preventing overexpression of BM components may delay, or even prevent, the development of the vascular lesions characteristic in diabetic retinopathy. However, our report [6] showed that the implementation of an antisense oligonucleotide strategy for the treatment of diabetic retinopathy would require multiple intravitreal injections, thus making it impractical for a long-term complication such as diabetic retinopathy. A siRNA strategy has been reported to have a longer duration of efficacy than the antisense oligonucleotides [8], so we tested its long-term efficacy in preventing BM overexpression and thickening in the retinal capillaries of diabetic rats.

In this study, we selectively targeted FN since previous studies indicated that downregulation of FN expression significantly reduces retinal capillary BM thickening and prevents retinal vascular lesions characteristic of diabetic retinopathy [6]. The siRNA approach effectively sustains a reduction in high glucose-induced FN overexpression in microvascular endothelial cells in cultures [9], and FN-siRNA exhibits long-term efficacy in downregulating FN overexpression in vitro. Therefore in this study, we investigated the in vivo efficacy of FN-siRNA as a long-term modulatory strategy by assessing its safety and tolerability; its localization in targeted retinal vascular cells; its ability to reduce specific gene (FN) expression on a long-term basis in retinal vascular cells; and its ability to prevent the development of acellular capillaries and pericyte loss in diabetic rats, two prominent vascular lesions characteristic of diabetic retinopathy.

## Methods

### Dose response study of intravitreally injected FN-siRNA

Different concentrations of FN-siRNA (0.5, 1, 3, or 6 µM) were tested for 1 week to identify an effective dose for reducing FN expression by approximately 30%. The target for achieving 30% downregulation was selected based on our previous studies, which is consistent with the magnitude of the increase caused by diabetes. As such, a 30%–50% decrease in FN expression would bring diabetic levels to a normal range [10].

### Time course study of intravitreally injected FN-siRNA

To determine the duration efficacy of the FN-siRNA strategy in reducing FN expression, retinas of rats intravitreally injected with 3 μM of FN-siRNA were studied at different time points (3 days, 1, 2, 3, and 6 weeks). The retinal proteins from rats sacrificed at these time points were analyzed for their FN protein level, which was then compared to that of uninjected control rats.

### Streptozotocin-induced diabetic rats and FN-siRNA intravitreal injection

In this study, 32 male Sprague Dawley rats were randomly assigned into four groups: normal, diabetic, diabetic injected with FN-siRNA, and diabetic injected with scrambled siRNA. Diabetes was induced using streptozotocin (STZ) as previously described [7]. SiRNA was administered intravitreally every six weeks over the study period of six months, for a total of three injections. Intravitreal injections were performed behind the ora serrata in the pars plana, directly into the vitreal cavity. Intravitreal injections were performed at an acute angle of about 30°, with a 30 gauge needle. The penetrance of the needle during the intravitreal injection was about 1 mm. Ten µl of freshly prepared 3 µM siRNA was injected every six weeks; that is, at the 1.5, 3, and 4.5 month time points. At the end of six months, all animals were sacrificed by CO_2_ inhalation as described and approved by the Boston University School of Medicine animal care board. No untoward effects were noted from the intravitreal injections in any of the eyes, as there was no redness or opacity as determined through routine eye examinations, as previously tested [11]. One eye from each animal was used for western blot analysis to determine the FN protein level, while the contralateral eye was subjected to retinal trypsin digestion (RTD) to isolate the retinal vasculature. These isolated retinal vasculatures were then stained with hematoxylin and periodic acid-Schiff (PAS) and were analyzed for retinal vascular lesions. Additionally, dose response and time course studies were performed using four animals for each dose or time point. Blood glucose, bodyweight, and HbA1c levels were monitored throughout the study and insulin injections were administered as required to maintain bodyweight and avoid ketoacidosis.

### Localization of FN-siRNA in retinal vascular cells

The 21-mer FN-siRNA (5′-AAC TTC AAA TTA TGA ACA AGA-3′) used in this study was designed on the basis of Tuschl’s rules (AAC19 adjacent nucleotides) [12] and encompassed the translation initiation site of the FN transcript as described. It was previously tested in vitro for its efficacy against FN overexpression [9]. To avoid any length-related nonspecificity, we used non-specific scrambled siRNA, niR16 of a similar length (5′-AAU AUU GGC GUU AAG AUU CUA- 3′), as a control in this study. Localization and distribution of FN-SiRNA in rat retinas was assessed by intravitreally injecting Cy3-labeled FN-siRNA and observing localized fluorescence in rat retinal sections at different time points (2 h, 6 h, and 4 days) post intravitreal injection. Retinal sections from rat eyes injected with Cy3-labeled scrambled siRNAs exhibited a similar distribution pattern to those of FN-siRNA at all time points observed. However, the biologic effect of the scrambled siRNA compared to that of the FN-siRNA was markedly different, with practically no effect on FN expression in rat retinas.

### Electron microscopy

Retinas isolated from enucleated eyes were immediately fixed in 2.5% glutaraldehyde in a 0.1 M cacodylate buffer. The tissues were dehydrated using osmium tetraoxide, ethanol, and propylene oxide (EMS, Hatfield, PA). The tissues were then embedded in an Epon-Araldite plastic mixture and baked for 48 h. Ultra-thin sections were serially cut at 60–70 nm using a microtome (LKB Ultratome Nova, Bromma, Sweden). The sections were collected and placed on a copper grid and stained with 4% uranyl acetate (EMS) in methanol and viewed under a transmission electron microscope (Philips, Electron Optics, Eindhoven, Netherlands). At least ten random images of retinal capillaries from the outer plexiform and ganglion cell layers from each individual animal were photographed and enlarged to 50,000× magnification for examination. These electron micrographs were analyzed according to the orthogonal intercept method [13] for BM thickness.

### Measurement of retinal capillary basement membrane thickness and retinal histopathology

Retinal capillary BM thickness was determined from electron micrographs using the Siperstein method [13]. In brief, using a computer with Adobe Systems Inc., San Jose, CA, a 20-spoke radial grid was superimposed over each capillary micrograph, and the thickness of the BM was measured at the point where it intersected with a spoke. The widths of the two thinnest portions of the BM surrounding each capillary were also measured. Most BM width measurements were performed on capillaries from the outer plexiform layer of the mid retina. At least ten capillaries were photographed and measured from each of the ten retinal sections per animal to determine the capillary BM thickness. Areas of the BM in which pericytes overlapped with endothelial cells were excluded from the analysis. Data was collected and evaluations were performed in a masked manner to hide the identity of the rats.

### Isolation and assessment of pericyte loss and acellular capillaries

To analyze the retinal vasculature for pericyte loss and acellular retinal capillaries, we used the retinal trypsin digest (RTD) technique as described by Kuwabara and Cogan [14] with minor modifications. Briefly, after enucleation, eyes were fixed in 10% formalin, intact retinas were dissected out and subjected to 3% trypsin digestion at 37 °C for 2–3 h with gentle shaking. Under the dissecting microscope, the nonvascular mass was removed from the entire vascular network and was then mounted and stained with PAS (Sigma-Aldrich, St. Louis, MO) and hematoxylin. The slides were immersed in 0.5% periodic acid (Sigma) for 10 min, rinsed in water, and reacted in Schiff’s reagent (Sigma) for 10 min. After a water rinse, the slides were dipped in acid ethanol (1% HCl, Sigma), rinsed, and reacted in 1% LiCO_3_ (Sigma). After dehydration in ethanol and clearing in xylene, the slides were mounted with Permount. The PAS stained the glycoprotein of the BMs pink, while the cellular nuclei (elliptical and oriented along the circumference of the capillary cross-section for endothelial cells; round in shape and abutting the outer portion of the capillary wall for pericytes) were stained blue by the hematoxylin. Representative areas of the vascular network were photographed using a Nikon DS-Fi1(Nikon Instruments, Melville, NY). The images were analyzed for retinal morphology and in particular for pericyte loss and acellular capillaries.

### Estimation of pericyte loss and acellular capillaries

Approximately 1,200 capillary cells were counted from the midretinal area of each retina following RTD and hematoxylin-PAS staining. At first, the images of retinal capillaries were captured using a Kodak digital camera connected to a computer and then pericyte counts were determined from the images. The specific loss of pericytes could be detected from the empty “shell” left behind after pericyte “dropout.” Capillaries that lacked both pericytes and endothelial cells were considered acellular. A defined area (1 mm^2^) was analyzed for each of the treated and untreated groups to compare the number of acellular capillaries.

### Statistical analysis

**Figure 1:**
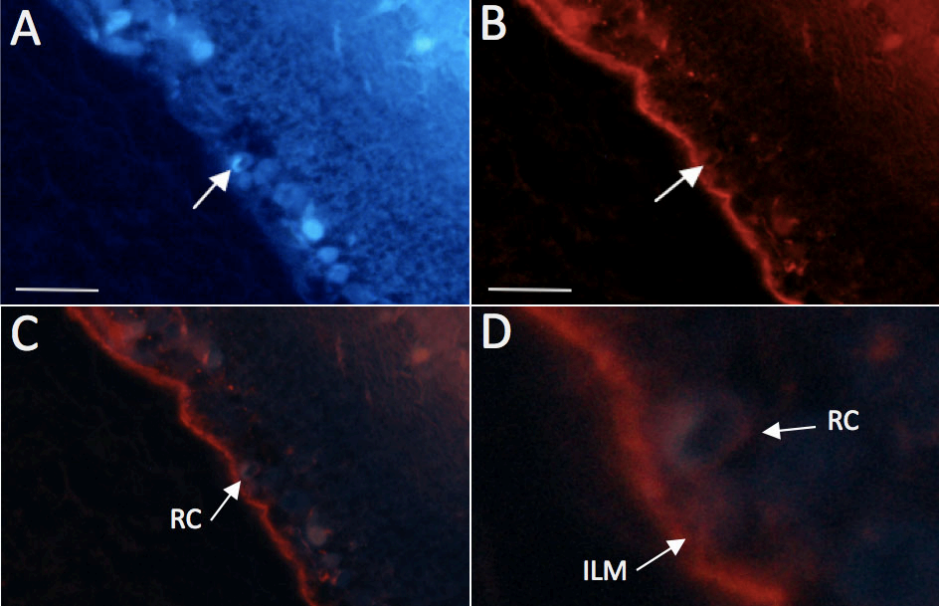
Localization of intravitreally injected fibronectin (FN)-SiRNA in vascular cells of a rat retina. FN-siRNA injected intravitreally in rat eyes localized on the inner limiting membrane and in retinal microvessels. The arrow points to a microvessel showing an endothelial cell nucleus (4',6-diamidino-2-phenylindole [DAPI]-positive) in panel **A**, and panel **B** shows Cyanine-3 labeled FN-siRNA localized in the same endothelial cell of the retinal microvessel. Panel **C** shows a superimposed image of **A** and **B**. Panel **D** shows a close-up view of the retinal microvessel shown in panel **C**. Bar: 50 μm. Inner limiting membrane (ILM); Retinal capillary (RC).

**Figure 2:**
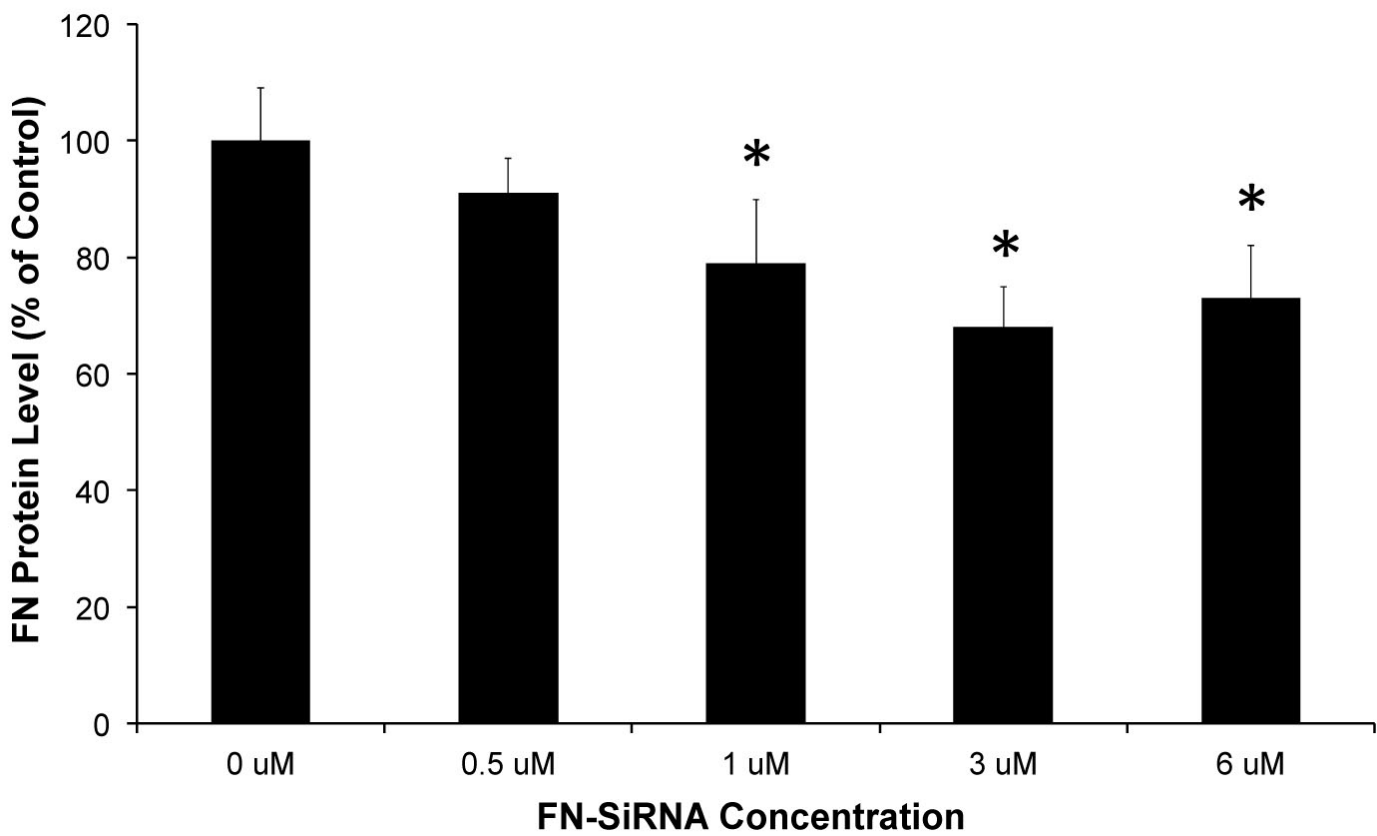
Dose response study showing fibronectin (FN)–siRNA efficacy. Different doses of FN-siRNA were tested at a 1-week time point to identify an optimal dosage for effective downregulation of FN expression. The graph shows reduced FN levels in rat retinas after intravitreal injection with increasing concentrations (0.5–6.0 μM) of FN–SiRNA. A 3 μM dose was most effective at downregulating retinal FN protein expression. *=p<0.05 versus “0 μM” group mean; n=4/group.

**Figure 3:**
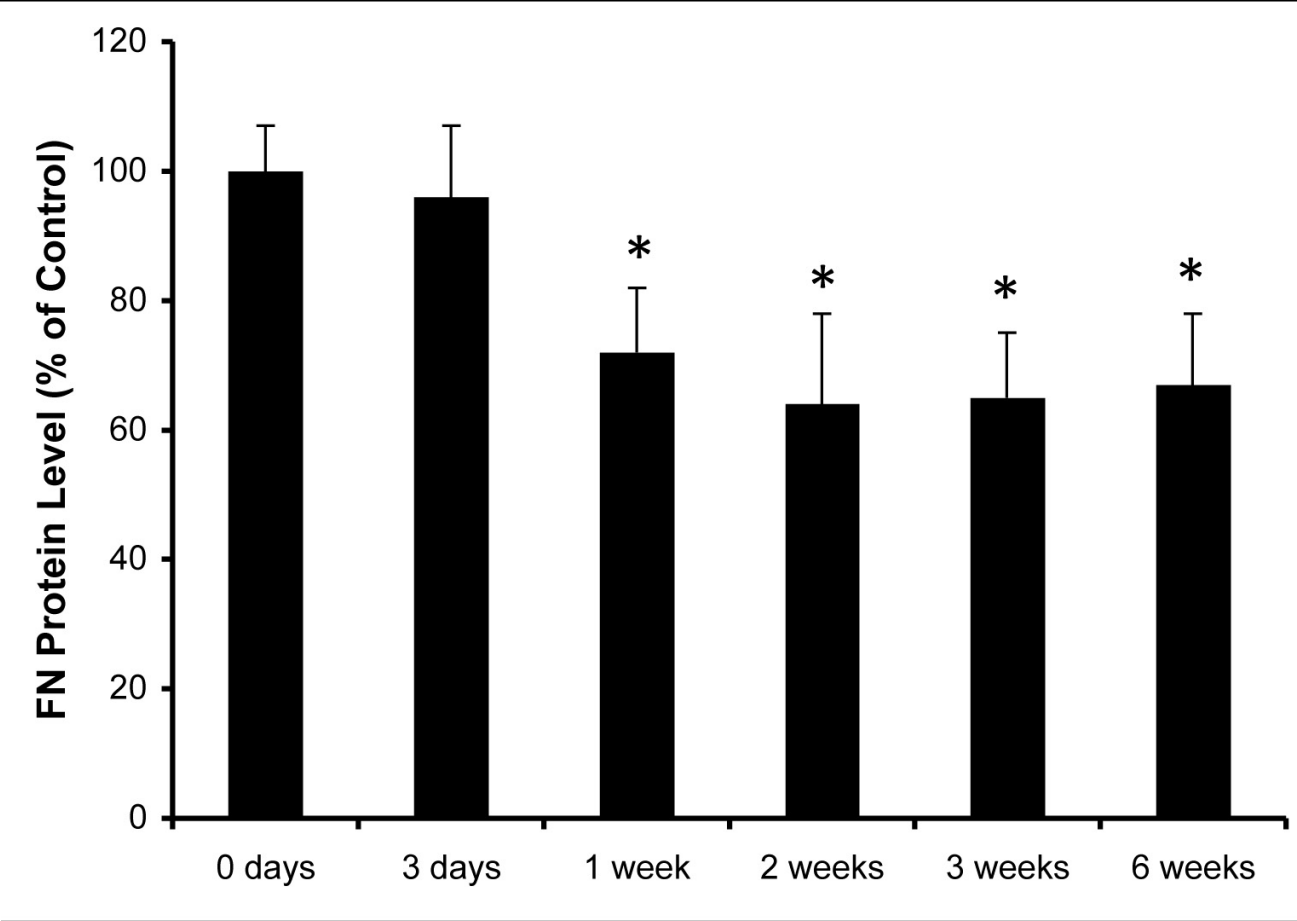
Time course study showing fibronectin (FN)–SiRNA efficacy. Relative levels of FN protein in rat retinas at different time points (3 days–6 weeks) after intravitreal injection of 3 μM FN–siRNA. By the 1-week time point, retinal FN protein expression was significantly reduced and remained inhibited at the 6-week time point. Scrambled siRNA showed no effect on the FN protein level. *=p<0.05 versus “0 days” group mean; n=4/group.

**Figure 4:**
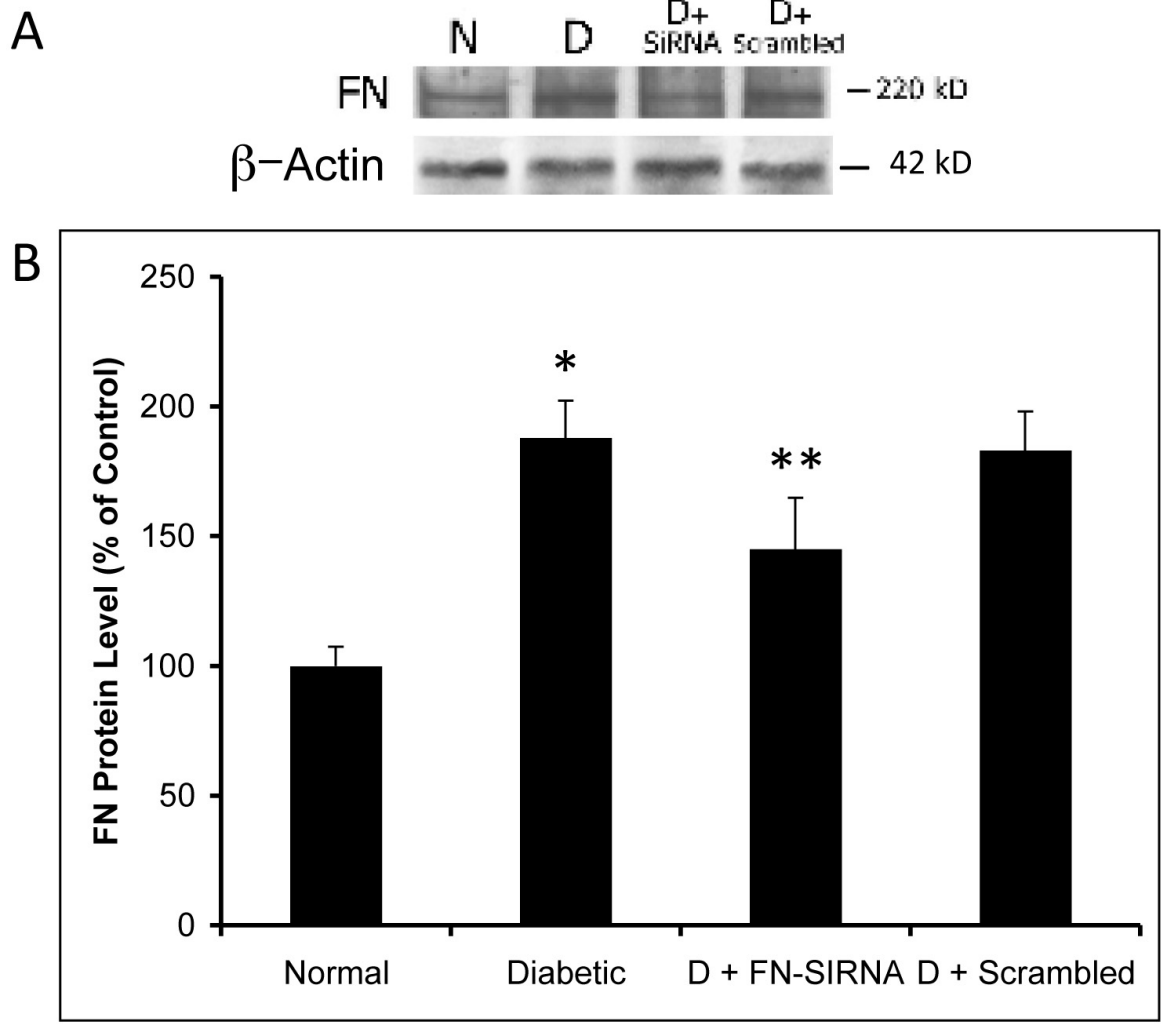
Effect of fibronectin (FN)-siRNA on retinal FN protein expression. **A**: Representative western blot image with an internal loading control β–actin showing the effect of FN-siRNA on retinal FN protein expression in diabetic rats. FN-siRNA treatment in the diabetic rats specifically reduced retinal FN protein expression compared to that in uninjected diabetic rats. **B**: Graphical representation of retinal FN protein expression in four groups of rats—normal, diabetic, diabetic injected with FN-siRNA, and diabetic injected with scrambled siRNA—showing that an intravitreal injection of FN-siRNA significantly reduces FN protein expression in diabetic rat retinas. Normal versus Diabetic *=p<0.05; Diabetic versus Diabetic+FN-siRNA **=p<0.05, n=8.

**Figure 5:**
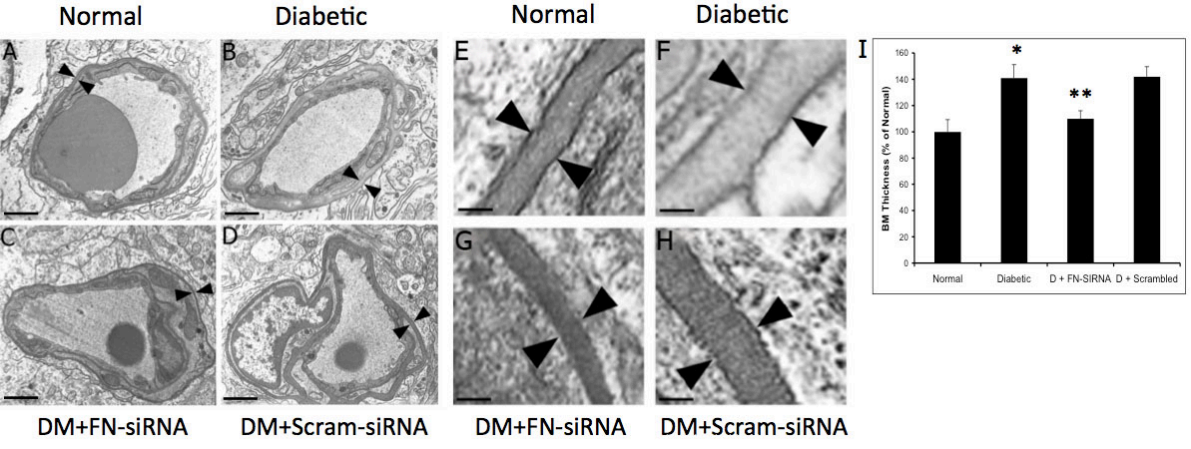
Electron microscopy (EM) analysis of capillary basement membrane (BM) thickening in rat retinas. **A**-**D**: Effect of fibronectin (FN)-siRNA on retinal capillary BM thickening in diabetic rats. Representative EM images of transverse sections of retinal capillaries of (**A**) normal rat, (**B**) diabetic rat, (**C**) diabetic rat intravitreally injected with FN-siRNA, and (**D**) diabetic rat intravitreally injected with scrambled siRNA; scale bar=1 μm. Enlarged views: **E**-**H**; scale bar=100 nm. FN-siRNA treatment in the diabetic rats specifically prevented BM thickening (arrowheads) compared to uninjected diabetic rats. (**I**) Graphical representation of retinal vascular BM thickness in four groups of rats: normal, diabetic, diabetic injected with FN-siRNA, and diabetic injected with scrambled siRNA, showing that an intravitreal injection of FN-siRNA reduces retinal capillary BM thickening in diabetic rats. Normal versus Diabetic *=p<0.05; Diabetic versus Diabetic+FN-siRNA **=p<0.05, n=8.

**Figure 6:**
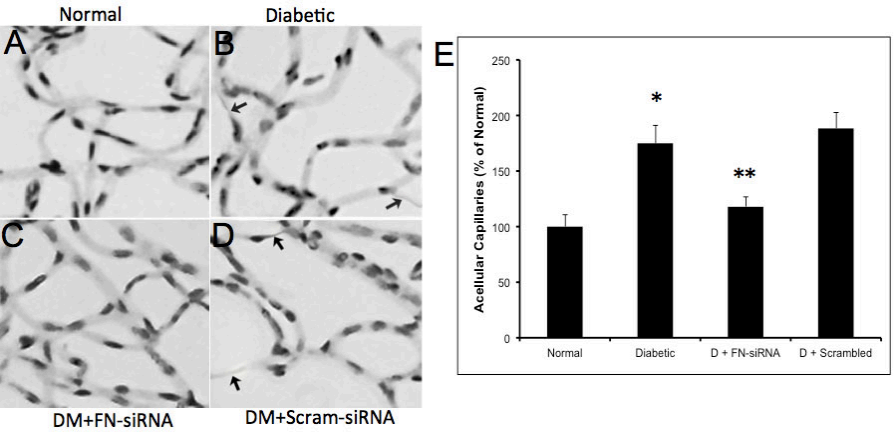
Development of acellular capillaries in the diabetic retina. **A**-**D**: Representative retinal trypsin digest (RTD) images showing the effect of fibronectin (FN)-siRNA on the development of acellular capillaries (as indicated by arrows) in retinas of a (**A**) normal rat, (**B**) diabetic rat, (**C**) diabetic rat intravitreally injected with FN-siRNA, and (**D**) diabetic rat intravitreally injected with scrambled siRNA. FN-siRNA treatment in the diabetic rats specifically reduced the development of acellular capillaries compared to those of uninjected diabetic rats. **E**: Graphical representation of acellular capillaries in four groups of rats: normal, diabetic, diabetic injected with FN-siRNA, and diabetic injected with scrambled siRNA, showing that an intravitreal injection of FN-siRNA reduces the development of acellular capillaries in diabetic rat retinas. Normal versus Diabetic *=p<0.05; Diabetic versus Diabetic+FN-siRNA **=p<0.05, n=8.

**Figure 7:**
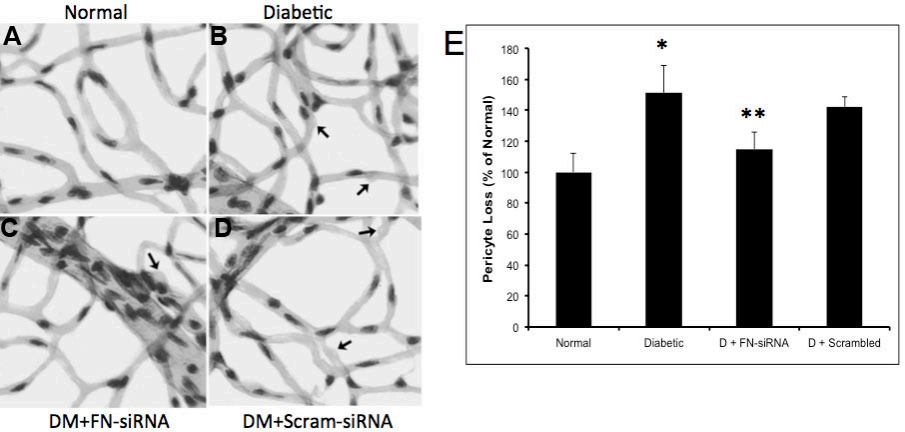
Retinal pericyte loss in diabetic rats. **A**-**D**: Representative retinal trypsin digest (RTD) images showing the effect of fibronectin (FN)-siRNA on the development of pericyte ghosts (as indicated by arrows) in retinas of a (**A**) normal rat, (**B**) diabetic rat, (**C**) diabetic rat intravitreally injected with FN–siRNA, and (**D**) diabetic rat intravitreally injected with scrambled siRNA. FN-siRNA treatment in the diabetic rats specifically reduced pericyte loss compared to uninjected diabetic rats. **E**: Graphical representation of pericyte dropouts in four groups of rats—normal, diabetic, diabetic injected with FN-siRNA, and diabetic injected with scrambled siRNA—showing that an intravitreal injection of FN-siRNA reduces the pericyte loss in diabetic rat retinas. Normal versus Diabetic *=p<0.05; Diabetic versus Diabetic+FN-siRNA **=p<0.05, n=8.

The data are reported as the means±SD. Dunnett’s comparison was only used against the control to analyze the data presented in Figure 1, Figure 2, and Figure 3. One-way ANOVA followed by a Student–Newman–Keuls test was used to analyze data in Figure 4, Figure 5, Figure 6, and Figure 7. Data with values of p<0.05 were considered significant.

## Results

### Localization of FN-SiRNA in rat retinal vascular cells after intravitreal injection

Retinal sections of the intravitreally injected Cy3-labeled FN-siRNA performed together with 4',6-diamidino-2-phenylindole (DAPI) were examined using confocal microscopy. FN-siRNA localization in the retinal vascular cells of the rat eyes was observed within 2 h post-injection. Based on the double stain from Cy3 and DAPI, fluorescence from the siRNA could be assigned to the nuclei of retinal vascular endothelial cells and pericytes (Figure 1A-D). The luminally located endothelial cell was distinguished from the abluminally located pericytes by its elongated nucleus.

### Dose response study showing FN-SiRNA efficacy

The effectiveness of the FN-siRNA strategy was tested using increasing concentrations (0.5–6.0 µM) of siRNA to identify an optimal dose for ~30% downregulation of FN expression. A reduced FN level was observed in rat retinas after intravitreal injection at concentrations ≥1 μM at 1 week post-injection (Figure 2). The 1 µM, 3 µM, and 6 µM dose significantly reduced FN expression (79±11% of control, p=0.03; 68±7% of control, p=0.01; 73±9% of control, p=0.02, respectively).

### Time course study showing FN-SiRNA efficacy

A time course (0–6 weeks) study following intravitreal injection of FN-siRNA showed significant downregulation of FN expression in the retinas. After 1 week, retinal FN expression was reduced by approximately 35%, which persisted to the 6 week time point (Figure 3). Scrambled siRNA showed no effect on the retinal FN protein level at any time point and showed no significant difference between the control (uninjected eyes) and the eyes injected with FN-siRNA.

### Effect of FN-SiRNA on retinal FN protein level

Western blot analyses indicated a significant increase in FN expression in the retinas of diabetic rats compared to those of non-diabetic control rats (188±14.2% of control versus 100±7.4% of control, p<0.002), respectively. A significant downregulation in FN expression was observed in the retinas of diabetic rats that were intravitreally injected with FN-siRNA compared to those of uninjected diabetic control eyes or those of diabetic eyes injected with scrambled siRNA (145±19.9% of control versus 188±14.2% of control, p=0.03; 145±19.9% of control versus 183±15% of control, p=0.03, respectively; Figure 4A,B).

### Effect of reduced FN on retinal capillary basement membrane thickness

An electron microscopy analysis showed a significant increase (~40%) in retinal capillary BM thickness in the diabetic rats compared to those of non-diabetic control rats (72.5±5.0 nm versus 51.5±4.8 nm, p<0.001). Upon intravitreal injection with FN-siRNA, a significant reduction (~75%) in retinal capillary BM thickness was observed in the retinas of diabetic rats compared to those of uninjected control diabetic rats and diabetic rats intravitreally injected with scrambled siRNA (56.4±2.8 nm versus 72.5±5.0 nm, p=0.03; 56.4±2.8 nm versus 73.7±3.9 nm, p<0.01, respectively; Figure 5A-I).

### Effect of FN-SiRNA on the development of vascular lesions

In this study, we assessed two characteristic lesions of diabetic retinopathy—pericyte loss and acellular capillaries—both of which showed significant improvement in retinas treated with FN-siRNA. The increased pericyte loss (151±18%, p=0.02 of the control) and number of acellular capillaries (175±16%, p=0.01 of the control) observed in diabetic retinas was significantly reduced in retinas of diabetic rats treated with FN-siRNA (pericyte loss: 115±11%, p=0.02 of the control; acellular capillaries: 118±9%, p=0.01 of the control). The number of acellular capillaries (Figure 6A-E) and the pericyte loss (Figure 7A-E) in diabetic rats that received scrambled siRNA did not differ from those of the diabetic rats. There was a strong correlation between decreased pericyte loss and reduced BM thickening (r=0.71), as well as between a decreased number of acellular capillaries and reduced BM thickening (r=0.82).

### Efficacy and safety of long-term application of FN-SiRNA

Retinas injected with siRNA and examined 1, 2, 4, and 6 weeks post-injection showed no physical evidence of redness or opacity in the eyes, and retinal capillaries and sections examined in parallel showed no infiltration of macrophages.

### Blood glucose, bodyweight, and HbA1c levels

**Figure 8:**
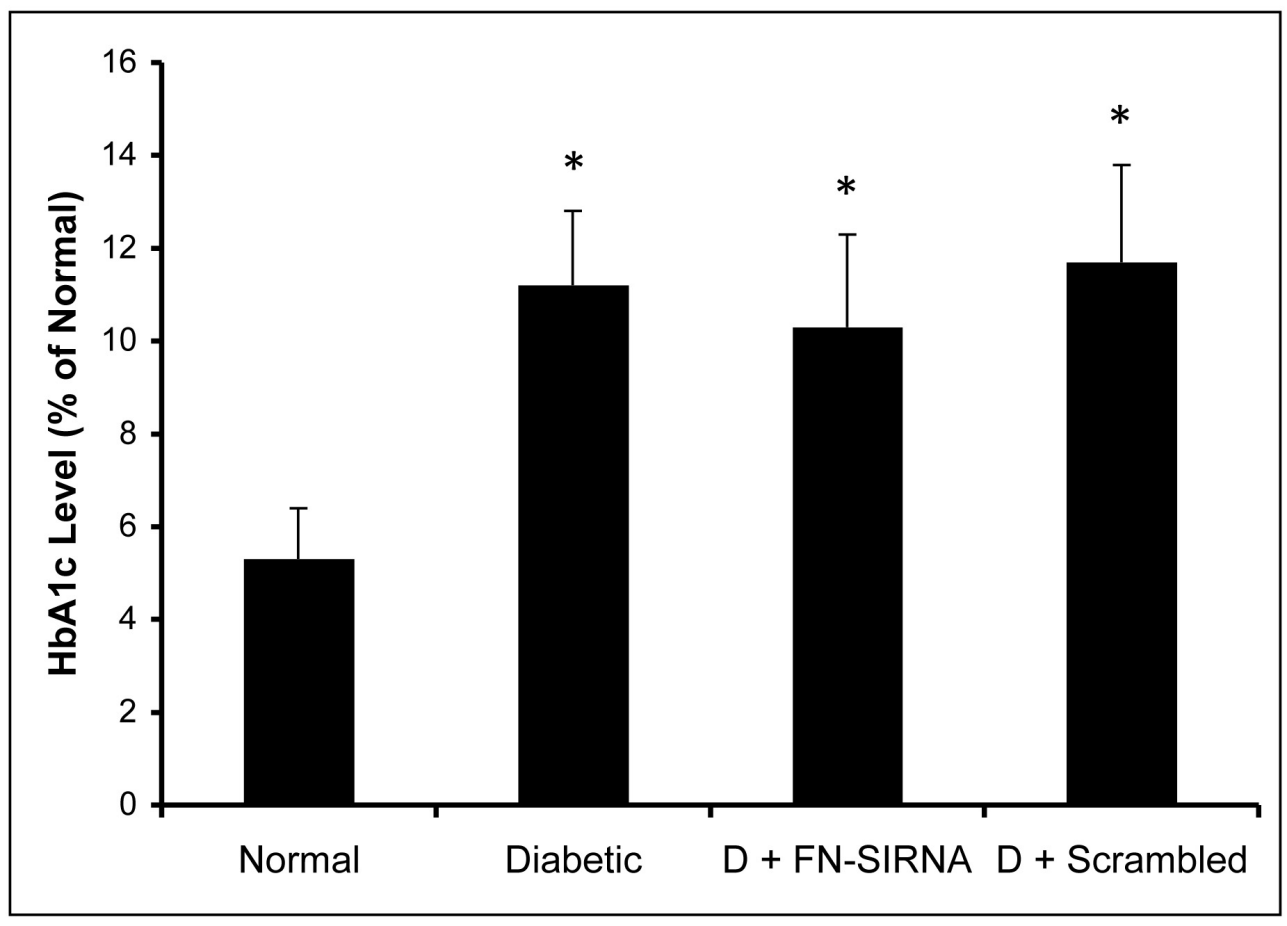
HbA1c levels in experimental animals. HbA1c levels measured at the end of the study confirmed hyperglycemic status in diabetic animals. Diabetic animals injected with fibronectin (FN)-siRNA or scrambled siRNA also displayed a similar hyperglycemic condition. *=p<0.05 versus “Normal” group mean; n=8/group.

Fasting blood glucose levels were measured every alternate day and showed hyperglycemic conditions in diabetic animals (307±48 mg/dl) compared to normal animals (122±21 mg/dl). The HbA1c level measured at the three-month interval also confirmed a hyperglycemic state in diabetic animals (11.2±1.6%) versus normal animals (5.3±1.1%; Figure 8). A reduced bodyweight was observed in the diabetic animals, which indicates a diabetic condition in these animals.

## Discussion

In this study, we demonstrated that the FN-siRNA approach is effective in downregulating overexpression of FN, a BM component, and that it resulted in beneficial effects, including a significant reduction of vascular BM thickening in the diabetic retina. The results from this study suggest that hyperglycemia-induced overexpression of FN plays a critical role in the development of capillary BM thickening in experimental diabetic animals. This study reports that an FN-siRNA strategy is effective in preventing hyperglycemia-induced BM thickening in retinal capillaries. To our knowledge, no study has yet been conducted that examines the efficacy of a siRNA strategy in reducing capillary BM thickening in the diabetic retina and its subsequent effects.

In the present study, we demonstrated that overexpression of FN in retinal vascular cells of diabetic rats was inhibited by intravitreal injection(s) of FN-siRNA, which resulted in a reduction in BM thickness and other beneficial outcomes, including the prevention of pericyte loss and acellular capillaries. This finding was consistent with our previous results demonstrating that reducing overexpression of BM components can decrease BM thickening in retinal capillaries [6]. The present results showed that inhibition of BM thickening using a siRNA strategy prevented retinal vascular cell loss, thus suggesting that overexpression of BM components promotes BM thickening and cell loss, and that FN-siRNA is an effective preventative strategy. Although it is unclear how BM thickening promotes cell loss, our previous report suggested that the extracellular matrix (ECM) produced by endothelial cells grown in high glucose condition promotes apoptosis [15].

BM is a highly organized structure composed of multiple ECM components linked to each other in an orderly manner. The ordered structure appears to be compromised in the thickened BMs of diabetic retinal capillaries, as evidenced by altered ultrastructures such as a “Swiss cheese-like” appearance. These altered structures are at least in part due to a disturbed ratio among the ECM components – a likely consequence of the abnormal increase in synthesis of the ECM components [6,10,16–24]. Our finding that BM thickening is ameliorated by FN-siRNA suggests that a siRNA strategy could be useful in preventing vascular abnormalities associated with diabetic retinopathy.

One mechanism responsible for triggering apoptotic signaling is the high glucose- or diabetes-induced forkhead box protein 1 (FOXO1) activation [25]. A previous study indicates that upon cell-matrix adhesion, beta-1 integrin can trigger the activation of the PI3K/Akt pathway, which then leads to the displacement of FOXO1 from a target promoter [25–27]. Clearly, communication between cells and the thickened BM is hampered, which can result in the promotion of apoptosis.

Previously, we identified a FN-siRNA that exhibits a long-term effect in microvascular endothelial cells, in vitro [9]. Time course studies with the same siRNA conducted in rat retinas indicated a significant decrease in FN expression that lasted for six weeks. We also determined the long-term efficacy of a FN-siRNA strategy—over a six month period with injections at 1.5, 3, and 4.5 months—in preventing characteristic FN overexpression and retinal vascular lesions in retinas of diabetic rats. This regimen provides a 50% improvement over a monthly antisense oligonucleotides regimen. In the long term, the benefits include a significant reduction in the number of injections. The results from this study suggest that the siRNA approach may have a potential long-term effect against the development of vascular lesions associated with diabetic retinopathy. Moreover, the consistent finding that FN-siRNA was efficacious, while scrambled siRNA had no effect, suggests that the results were not due to non-specific effects.

The FN siRNA used in this study was specifically targeted to the FN translation initiation site of the FN transcript, with the goal of reducing hyperglycemia-induced FN overexpression by 50%. Our studies indicate that an approximate 50% FN reduction was achieved using the 21-mer FN siRNA. However, it is possible that the efficacy may be increased if longer (25–30 mer) siRNAs [28] are used. The possibility of off-target effects may exist when siRNAs are used. However, in this study, the 21-mer FN siRNA suited the purpose and no off-target effects were detectable.
